# From antenatal contact to facility-based delivery: a provincial analysis of dropout along the maternal continuum of care in Afghanistan using the 2022–23 MICS

**DOI:** 10.1080/16549716.2026.2750043

**Published:** 2026-10-20

**Authors:** Rajeev Ranjan, Palwasha Anwari, Abigail Colson, Robert van der Meer

**Affiliations:** a Department of Management Science, University of Strathclyde, Glasgow, UK; b Department of Health Services Research and Policy, London School of Hygiene & Tropical Medicine, London, UK

**Keywords:** Skilled birth attendance, health service utilisation, subnational inequality, reproductive health, survey-weighted analysis

## Abstract

**Background:**

Although 77.7% of ever-married women with a recent live birth in Afghanistan attended at least one antenatal care (ANC) contact, 74.5% of ANC-attending women delivered in a health facility. Provincial variation in this transition remains poorly characterised.

**Objective:**

To examine associations of ANC contact frequency and measured clinical content with facility-based delivery and to characterise provincial patterns of continuity.

**Methods:**

We analysed the Afghanistan Multiple Indicator Cluster Survey 2022–23. The cohort included 9,268 ANC-attending ever-married women aged 15–49 years. Survey-weighted logistic regression examined ANC contact frequency (1–3, 4–7, ≥8) and a three-item measured clinical-content score (0–3). Province-specific ANC 4+ coverage and facility-based delivery were also examined.

**Results:**

Facility-based delivery was 74.5%. Compared with 1–3 contacts, adjusted odds were higher with 4–7 contacts (adjusted odds ratio 1.49; 95% confidence interval 1.25–1.77) and ≥8 contacts (adjusted odds ratio 1.93; 95% confidence interval 1.45–2.57), corresponding to adjusted probability differences of 6.5 and 10.3% points. For measured clinical content, a score of 3 versus 0 was associated with a 4.6-point higher adjusted probability, while intermediate scores showed no clear gradient. Provincial facility-based delivery ranged from 16.8% to 93.3%, with substantial heterogeneity in joint ANC and delivery patterns.

**Conclusions:**

Among women who had initiated ANC, approximately one quarter delivered outside a health facility. Greater ANC contact frequency was consistently associated with facility-based delivery, while marked provincial variation highlights the importance of continuity between antenatal and childbirth care.

## Paper Context

**Main findings**: Among ever-married women with a recent live birth who had attended antenatal care, approximately one quarter did not subsequently deliver in a health facility, and higher antenatal contact frequency was associated with greater facility-based delivery.

**Added knowledge**: Province-level analysis showed heterogeneous joint patterns of antenatal care depth and facility-based delivery, with substantial uncertainty around several median-based classifications.

**Global health impact for policy and action**: Maternal health programmes in fragile settings should consider continuity after antenatal care initiation, alongside barriers to childbirth care, and use province-specific estimates with their uncertainty rather than national averages alone.

## Background

Afghanistan continues to face one of the highest maternal mortality burdens globally, with an estimated maternal mortality ratio of 521 deaths per 100,000 live births in 2023 [1]. Despite progress in maternal health indicators over the past two decades, access to and use of essential maternal health services remain uneven across geographic and socioeconomic groups [2–4]. Facility-based delivery with skilled attendance is an important component of maternal care because timely access to qualified personnel, referral, and emergency obstetric services can reduce preventable maternal and neonatal complications [5]. Yet national coverage of maternal services remains incomplete and masks substantial inequalities between urban and rural areas and across provinces [3,6].

The Afghanistan Multiple Indicator Cluster Survey (MICS) 2022–23, the first nationally representative household survey conducted after the 2021 political transition, highlights an important discontinuity in the maternal care pathway [7]. Among ever-married women with a recent live birth, 77.7% attended at least one antenatal care (ANC) contact. Among the subset who attended ANC, 74.5% subsequently delivered in a health facility. These percentages have different denominators: the first describes initiation of ANC among women with a recent birth, whereas the second describes facility-based delivery conditional on prior ANC attendance. The latter therefore identifies a specific continuity-of-care question – why a substantial proportion of women who have already entered maternal healthcare during pregnancy do not subsequently use a health facility for childbirth.

Most studies in Afghanistan and comparable low- and middle-income settings examine determinants of facility-based delivery in the broader population and consistently identify household wealth, maternal education, rural residence, geography, and access as important correlates [6,8,9]. Such analyses are essential for understanding overall utilisation, but they combine at least two analytically distinct processes: failure to enter maternal healthcare during pregnancy and failure to continue after initial contact. Restricting the present analysis to women who had already attended ANC allows the second process to be examined directly, while recognising that the resulting estimates apply to a selected subgroup rather than to all women with a recent birth.

The World Health Organisation’s 2016 ANC model emphasises both repeated contact during pregnancy and the quality and content of care delivered during those contacts [10,11]. Repeated contacts can create opportunities for clinical monitoring, counselling, birth preparedness, recognition of danger signs, referral, and familiarity with health services. At the same time, the content of individual contacts matters for the clinical value of ANC. In MICS, selected aspects of content can be described through self-reported receipt of blood pressure measurement, urine testing, and blood testing. These indicators capture specific clinical services, but they do not represent comprehensive ANC quality, which also includes counselling, respectful care, communication, provider competence, continuity, and facility readiness [10,11].

ANC contact frequency and measured clinical content are therefore related rather than independent competing dimensions. Women with more contacts have more opportunities to receive the measured services, and measured content may partly lie on a pathway linking repeated engagement with subsequent care-seeking. We therefore examined separate and mutually adjusted associations, expressed contrasts on a common adjusted probability scale, and assessed whether associations varied across combinations of frequency and content. Evidence from other low- and middle-income settings also links greater ANC contact frequency with facility or skilled delivery, including eight or more contacts [12,13]. The World Health Organisation’s 2016 recommendations likewise formalised an eight-contact model for routine antenatal care [14].

This distinction is particularly relevant in Afghanistan, where the Basic Package of Health Services has historically sought to strengthen maternal healthcare through standardised service provision, provider training, supervision, and service availability [15]. At the same time, women’s ability to return repeatedly during pregnancy or reach a facility during labour is shaped by factors extending beyond the content of clinical encounters, including geography, transport, security, household decision-making, and the availability of functioning services [16–18]. These constraints vary substantially across the country. National averages may therefore conceal provinces in which ANC engagement and delivery care move together and others in which they diverge.

The present study focuses on continuity after ANC initiation. The objectives were: (i) to examine the separate and mutually adjusted associations of ANC contact frequency and measured clinical content with facility-based delivery among women who had initiated ANC, including whether these associations interacted; (ii) to characterise provincial variation in ANC 4+ coverage and facility-based delivery; and (iii) to describe how these indicators vary jointly across provinces and assess uncertainty surrounding the observed patterns. The provincial analysis was used to characterise geographic variation in patterns of ANC engagement and facility-based delivery across Afghanistan.

## Methods

### Study design and data source

This study is a secondary cross-sectional analysis of the Afghanistan MICS 2022–23, conducted by UNICEF in collaboration with the National Statistics and Information Authority. The survey used a multistage stratified cluster sampling design, with primary sampling units selected within province and urban/rural strata, and provided women-specific sampling weights for population-level estimation [7].

The women’s questionnaire collected reproductive and maternal health information from ever-married women aged 15–49 years, including ANC use, timing and selected content of care, place of delivery, and assistance at childbirth for the most recent live birth. The study is reported in accordance with the STROBE guidance for cross-sectional studies [19]. Standard MICS tools and questionnaires are described by UNICEF [20].

### Study population

Among 28,563 ever-married women aged 15–49 years, 12,578 had the relevant recent-birth maternal-care information and 9,268 reported at least one ANC contact for the most recent live birth. The study intentionally conditions on prior ANC attendance because the research question concerns continuation from ANC to delivery care rather than initiation of maternal healthcare. Accordingly, the findings apply specifically to women who had already attended ANC and may not be generalisable to all women with a recent birth.

One woman had an unclassifiable place-of-delivery response. Missing or non-substantive responses were also identified for ANC contact frequency (*n* = 18), timing of ANC initiation (*n* = 11), and at least one measured clinical-content component (*n* = 19); these categories overlap. Wealth, education, residence, maternal age, and parity were complete. Overall, 9,223 women contributed complete data to the fully adjusted model. Participant flow is summarised in Supplementary Figure S2.

### Outcome variable

The primary outcome was facility-based delivery for the most recent live birth. Government hospitals, health centres, health posts, public clinics, other public facilities, private hospitals, private clinics, private maternity homes, other private medical facilities, and facilities for which the respondent could not specify public or private status were classified as facility deliveries. Deliveries at the respondent’s home, another home, or a location recorded as ‘other’ were classified as non-facility deliveries. A no-response code was treated as missing. The MICS place-of-delivery variable did not contain a separate community-facility category.

### Exposure variables

ANC contact frequency was derived from the reported number of contacts and categorised as 1–3, 4–7, or ≥8 contacts. ‘Don’t know’ and no-response values were treated as missing rather than included in the ≥8 category.

Measured ANC clinical content was operationalised as a limited indicator of selected services rather than a comprehensive measure of ANC quality. Blood pressure measurement, urine testing, and blood testing were each coded as received or not received and summed to a score from 0 to 3. Because the relationship between this score and facility-based delivery was non-monotonic, the score was modelled categorically rather than as a continuous linear term.

Timing of ANC initiation was represented by a binary indicator for first-trimester initiation.

### Covariates

Prespecified covariates were maternal age, parity, household wealth quintile, maternal education, and urban/rural residence, based on prior literature on maternal healthcare utilisation [3,9]. Maternal age was modelled per 10-year increase and parity per additional child.

### Statistical analysis

#### Descriptive and provincial-level analysis

Unweighted sample counts and survey-weighted percentages were used to describe the ANC-attending cohort. Province-specific weighted estimates were calculated for ANC 4+ coverage and facility-based delivery. Pearson correlations were used to describe ecological relationships between provincial facility-based delivery and ANC 4+ coverage, each measured clinical component, and the mean clinical-content score. These ecological analyses were considered descriptive and were not used to infer individual-level associations.

### Multivariable regression analysis

Survey-weighted logistic regression was used to estimate associations with facility-based delivery, with variance estimation based on robust standard errors clustered at the primary sampling unit (PSU) level [21]. Crude and adjusted odds ratios with 95% confidence intervals are reported. The adjusted model included wealth quintile, education, residence, maternal age, parity, ANC contact frequency, first-trimester initiation, and measured clinical content.

Because repeated ANC contacts provide additional opportunities to receive clinical services, ANC frequency and measured clinical content were not interpreted as competing exposures. A directed acyclic graph summarising hypothesised relationships among frequency, measured content, facility-based delivery, measured covariates, pregnancy-risk proxies, and unmeasured care-seeking/access factors is provided in Supplementary Figure S1. Separate models examined ANC frequency and measured content before the mutually adjusted model, which was interpreted as estimating conditional associations rather than causal effects.

An additional model included interaction terms between categorical ANC frequency and measured clinical content; the interaction terms were assessed jointly with a Wald test. Standardised adjusted probabilities and absolute percentage-point differences were calculated for each ANC-frequency category relative to 1–3 contacts and each content category relative to score 0. Measured clinical content was considered a potential intermediate pathway, but formal mediation analysis was not undertaken because temporal ordering and potential mediator–outcome confounding could not be adequately established.

### Provincial quadrant analysis

For each of the 34 provinces, survey-weighted ANC 4+ coverage and facility-based delivery among ANC-attending women were estimated. Provinces were positioned according to whether each estimate was above or below the median across the 34 province-specific estimates. The medians were 40.4% for ANC 4+ coverage and 77.5% for facility-based delivery; classification used the corresponding unrounded values. These are descriptive medians across province-specific, survey-weighted estimates and are not national, population-weighted prevalence estimates. This descriptive positioning is consistent with the use of subnational coverage comparisons to characterise geographic heterogeneity in reproductive, maternal, newborn, and child health [22].

The resulting four descriptive quadrants were high ANC 4+/high facility-based delivery, high ANC 4+/low facility-based delivery, low ANC 4+/high facility-based delivery, and low ANC 4+/low facility-based delivery. Province-specific 95% confidence intervals for both indicators were calculated on the logit scale and back-transformed to probabilities. Classification stability was further assessed by bootstrap resampling that recalculated province-specific estimates, the two medians, and quadrant membership in each replicate; the proportion of replicates retaining the point-estimate quadrant was used as a classification-retention measure. Variances for the province-specific survey-weighted proportions were estimated by Taylor linearisation, treating primary sampling units as clusters within the MICS sampling strata; the resulting standard errors were transformed to the logit scale for interval construction, and the confidence limits were back-transformed to probabilities.

### Sensitivity analyses

To examine whether the association between high ANC frequency and facility-based delivery differed according to a limited pregnancy-risk proxy, an interaction was fitted between ≥8 ANC contacts and higher-risk status, defined as maternal age >35 years, maternal age <18 years, or parity >4. This analysis was interpreted as an assessment of effect modification by the proxy rather than as a test that could establish or exclude reverse causality. Adjusted probabilities were calculated for 1–3 and ≥8 ANC contacts within the lower- and higher-risk groups.

The main model was also repeated using skilled birth attendance as an alternative outcome. Skilled attendance was defined according to the Afghanistan MICS indicator TM.9 as delivery attended by a medical doctor or nurse/midwife [7]. This definition is consistent with the broader concept of skilled attendance used for Sustainable Development Goal indicator 3.1.2 [23]. Province-level quadrants were recomputed using skilled birth attendance to assess sensitivity to the outcome definition.

### Patient and public involvement

No patients or members of the public were involved in the design, conduct, or reporting of this secondary analysis.

## Results

### Sample characteristics

Of 28,563 ever-married women aged 15–49 years, 12,578 had the relevant recent-birth maternal-care information and 9,268 had attended at least one ANC contact. One woman had an unclassifiable place of delivery. Forty-five women were excluded from the fully adjusted complete-case model because of missing or non-substantive responses in one or more analysis variables, leaving 9,223 women for multivariable analysis. The ANC-attending cohort was distributed across 954 PSUs and 66 strata.

Among the 9,267 women with a classifiable delivery location, the weighted facility-based delivery rate was 74.5% and the corresponding non-facility delivery rate was 25.5%. Most women had no formal education (72.4%) and lived in rural areas (73.8%). Household wealth was distributed across quintiles, with 15.9% in the poorest and 22.0% in the richest quintile. Mean maternal age was 28.3 years (SD 6.5) and mean parity was 4.2 children (SD 2.7). Table 1 presents the complete distribution of weighted sample characteristics.

**Table 1.** Sample characteristics of ANC-attending ever-married women with a recent live birth, Afghanistan MICS 2022–23 (cohort *n* = 9,268).

Characteristic	n	Weighted %
***OUTCOME***		
Facility-based delivery	6,878	74.5%
Non-facility delivery	2,389	25.5%
***WEALTH QUINTILE***		
Poorest	1,559	15.9%
Second	2,033	19.8%
Middle	2,182	20.7%
Fourth	1,987	21.7%
Richest	1,507	22.0%
***EDUCATION***		
No education	7,061	72.4%
Primary	917	11.6%
Lower secondary	409	5.3%
Upper secondary	576	6.9%
Higher/university	305	3.9%
***RESIDENCE***		
Urban	1,638	26.2%
Rural	7,630	73.8%
***ANC FREQUENCY***		
1–3 visits	5,486	56.9%
4–7 visits	2,967	34.1%
8+ visits	797	9.0%
***MEASURED ANC CLINICAL CONTENT***		
Score 0	1,173	11.7%
Score 1	2,686	29.3%
Score 2	1,951	20.4%
Score 3	3,439	38.7%
***FIRST-TRIMESTER ANC***		
Yes	4,797	50.4%
No	4,460	49.6%
***CONTINUOUS CHARACTERISTICS***		
Maternal age, mean (SD), years	9,268	28.3 (6.5)
Parity, mean (SD), children ever born	9,268	4.2 (2.7)

Values in the n column are unweighted sample counts; percentages are survey-weighted estimates using the MICS women’s sample weights and therefore may not correspond exactly to crude count proportions. The delivery-outcome denominator is 9,267 because one response was unclassifiable; other variable-specific denominators vary slightly because missing or non-substantive responses were excluded.

ANC contact frequency was predominantly below the eight-contact level: 56.9% of women reported 1–3 contacts, 34.1% reported 4–7 contacts, and 9.0% reported ≥ 8 contacts. First-trimester ANC initiation was reported by 50.4% of women. For the measured clinical-content score, 11.7% received none of the three recorded components, 29.3% received one, 20.4% received two, and 38.7% received all three. The weighted mean score was 1.86 (SD 1.06).

### Facility-based delivery among ANC attendees

Among non-facility deliveries, 10.4% involved a doctor or nurse/midwife, 1.5% a community health worker, 35.4% a traditional birth attendant, 55.9% a relative or friend, and 2.5% no attendant. These categories are based on MICS multiple-response birth-attendant items and are not mutually exclusive; consequently, their percentages can sum to more than 100%. The proportion of non-facility births attended by a doctor or nurse/midwife also explains why skilled birth attendance was slightly higher than facility-based delivery.

### Individual-level associations with facility-based delivery

#### ANC contact frequency and measured clinical content

Weighted facility-based delivery increased across ANC-frequency categories, from 68.2% among women with 1–3 contacts to 81.8% among those with 4–7 contacts and 86.5% among those with ≥8 contacts. In the mutually adjusted model, women with 4–7 contacts had higher odds of facility-based delivery than those with 1–3 contacts (adjusted odds ratio [aOR] 1.49; 95% CI 1.25 to 1.77), and the association was larger for ≥8 contacts (aOR 1.93; 95% CI 1.45 to 2.57). The corresponding crude odds ratios were 2.10 (95% CI 1.81 to 2.43) and 3.04 (95% CI 2.29 to 4.04), respectively (Table 2).

**Table 2.** Crude and adjusted associations with facility-based delivery among ANC-attending women, Afghanistan MICS 2022–23 (complete-case *n* = 9,223).

Variable	Category (ref)	Crude OR (95% CI)	Adjusted OR (95% CI)	Adj. p
Wealth quintile	*Poorest (ref)*	1.00	1.00	—
	Second quintile	1.38 (1.13–1.68)	**1.32 (1.09–1.60)**	0.005
	Middle quintile	2.22 (1.80–2.75)	**1.84 (1.48–2.28)**	<0.001
	Fourth quintile	3.86 (3.07–4.85)	**2.65 (2.11–3.32)**	<0.001
	Richest quintile	11.32 (8.09–15.83)	**4.88 (3.51–6.78)**	<0.001
Education	*No education (ref)*	1.00	1.00	—
	Primary school	1.68 (1.28–2.19)	**1.25 (0.97–1.62)**	0.087
	Lower secondary	2.33 (1.63–3.32)	**1.58 (1.09–2.28)**	0.015
	Upper secondary	3.63 (2.48–5.32)	**1.80 (1.22–2.65)**	0.003
	Higher / university	15.57 (6.92–35.02)	**4.88 (2.13–11.17)**	<0.001
Residence	*Urban (ref)*	1.00	1.00	—
	Rural	0.22 (0.15–0.31)	**0.50 (0.35–0.71)**	<0.001
Maternal age	Per 10 years	0.93 (0.84–1.02)	**1.46 (1.22–1.73)**	<0.001
Parity	Per additional child	0.91 (0.89–0.93)	**0.90 (0.87–0.94)**	<0.001
ANC frequency	*1–3 visits (ref)*	1.00	1.00	—
	4–7 visits	2.10 (1.81–2.43)	**1.49 (1.25–1.77)**	<0.001
	8+ visits	3.04 (2.29–4.04)	**1.93 (1.45–2.57)**	<0.001
First-trimester ANC	*No (ref)*	1.00	1.00	—
	Yes	1.73 (1.49–2.01)	**1.23 (1.05–1.45)**	0.011
Measured ANC clinical content	*Score 0 (ref)*	1.00	1.00	—
	Score 1	0.79 (0.65–0.97)	**0.87 (0.72–1.06)**	0.176
	Score 2	1.11 (0.89–1.38)	**1.02 (0.81–1.28)**	0.859
	Score 3	1.93 (1.56–2.40)	**1.33 (1.06–1.66)**	0.012

Odds ratios and 95% confidence intervals are survey-weighted; variance estimation uses robust standard errors clustered at the primary sampling unit (PSU) level. The adjusted model includes all variables shown simultaneously. Reference categories are indicated as ‘ref’. Adjusted *p* values are shown. Measured ANC clinical content was modelled categorically because its association with facility-based delivery was non-monotonic.

On the standardised adjusted probability scale, 4–7 contacts versus 1–3 contacts corresponded to an absolute difference of 6.5% points (95% CI 3.8 to 9.3), while ≥ 8 versus 1–3 contacts corresponded to a 10.3-point difference (95% CI 6.3 to 14.2). These estimates provide a directly comparable scale for interpreting ANC-frequency contrasts alongside the clinical-content measure.

The association between measured clinical content and facility-based delivery was not monotonic across the four score categories. Relative to score 0, the adjusted odds ratios were 0.87 (95% CI 0.72 to 1.06) for score 1, 1.02 (95% CI 0.81 to 1.28) for score 2, and 1.33 (95% CI 1.06 to 1.66) for score 3. The corresponding adjusted probability differences were −2.3% points (95% CI − 5.7 to 1.0), +0.3 points (95% CI − 3.5 to 4.2), and +4.6 points (95% CI 0.9 to 8.2). Descriptively, the score was also positively related to contact frequency: mean measured content increased from 1.62 among women with 1–3 contacts to 2.12 among women with 4–7 contacts and 2.41 among women with ≥8 contacts (Supplementary Table S3).

The sequential models showed attenuation after mutual adjustment. Before conditioning on measured content, the adjusted odds ratio for ≥8 versus 1–3 contacts was 2.11 (95% CI 1.58 to 2.81), compared with 1.93 after content was included. For a content score of 3 versus 0, the corresponding estimate was 1.47 (95% CI 1.18 to 1.84) before conditioning on ANC frequency and 1.33 after frequency was included. There was no clear evidence of interaction between categorical ANC frequency and measured content (joint Wald test, *p* = 0.43).

### Socioeconomic and demographic correlates

Facility-based delivery showed a strong socioeconomic gradient. Compared with women in the poorest quintile, adjusted odds increased across wealth groups and were highest among women in the richest quintile (aOR 4.88; 95% CI 3.51 to 6.78). The crude richest-versus-poorest odds ratio was substantially larger (11.32; 95% CI 8.09 to 15.83), indicating that part of the unadjusted wealth gradient was shared with education, residence, ANC utilisation, and other included covariates.

Maternal education was also positively associated with facility-based delivery at higher levels. Relative to no education, primary schooling was not statistically significant after adjustment (aOR 1.25; 95% CI 0.97 to 1.62), whereas lower secondary education (aOR 1.58; 95% CI 1.09 to 2.28), upper secondary education (aOR 1.80; 95% CI 1.22 to 2.65), and higher/university education (aOR 4.88; 95% CI 2.13 to 11.17) were associated with higher odds of facility-based delivery.

Rural residence remained strongly associated with lower facility-based delivery after adjustment (aOR 0.50; 95% CI 0.35 to 0.71). Higher parity was also inversely associated with facility-based delivery (aOR 0.90 per additional child; 95% CI 0.87 to 0.94). Maternal age showed a different pattern after adjustment: the crude association per 10-year increase was close to null (OR 0.93; 95% CI 0.84 to 1.02), whereas the adjusted association was positive (aOR 1.46; 95% CI 1.22 to 1.73), particularly after accounting for parity and the other covariates. First-trimester ANC initiation was associated with higher facility-based delivery (aOR 1.23; 95% CI 1.05 to 1.45).

### Provincial variation and ecological associations

Facility-based delivery among ANC-attending women varied markedly across Afghanistan, ranging from 16.8% in Nuristan to 93.3% in Kabul. This spread was far greater than the difference between the national conditional estimate and the provincial median, underscoring the extent to which a single national percentage can conceal subnational heterogeneity. Full province-specific estimates and 95% confidence intervals are reported in Supplementary Table S1.

At the ecological level, provincial ANC 4+ coverage was positively correlated with facility-based delivery (*r* = 0.53, *p* = 0.001). By contrast, correlations between the three measured clinical components and facility-based delivery were weaker and not statistically significant: blood pressure measurement *r* = 0.11 (*p* = 0.54), urine testing *r* = 0.13 (*p* = 0.45), and blood testing *r* = 0.21 (*p* = 0.24). The correlation with the mean clinical-content score was similarly weak (*r* = 0.20, *p* = 0.26). These coefficients describe relationships between provincial coverage measures and should not be interpreted as corresponding individual-level associations.

### Provincial quadrants and classification uncertainty

Using the medians across the 34 province-specific estimates (ANC 4 + 40.4%; facility-based delivery 77.5%), the point-estimate classification contained 10 provinces in the high ANC 4+/high facility-based-delivery quadrant, 7 in the high ANC 4+/low facility-based-delivery quadrant, 7 in the low ANC 4+/high facility-based-delivery quadrant, and 10 in the low/low quadrant (Table 3). The high ANC 4+/low facility-based-delivery group comprised Badakhshan, Daykundi, Faryab, Herat, Laghman, Panjshir, and Takhar. The low ANC 4+/high facility-based-delivery group comprised Baghlan, Ghazni, Jawzjan, Kapisa, Khost, Kunar, and Maidan Wardak. The remaining provinces were distributed across the high/high and low/low quadrants as shown in Table 3.

**Table 3.** Median-based provincial quadrants of ANC 4+ coverage and facility-based delivery, Afghanistan MICS 2022–23.

Quadrant	n	ANC 4+	Facility-based delivery	Provinces
Type 1: High ANC 4+ / High FBD	10	Above median	Above median	Balkh†, Bamyan†, Kabul, Kunduz†, Logar†, Nangarhar†, Nimroz†, Paktika†, Parwan, Sar-e-Pul†
Type 2: High ANC 4+ / Low FBD	7	Above median	Below median	Badakhshan, Daykundi, Faryab†, Herat†, Laghman†, Panjshir†, Takhar†
Type 3: Low ANC 4+ / High FBD	7	Below median	Above median	Baghlan†, Ghazni†, Jawzjan†, Kapisa†, Khost, Kunar, Maidan Wardak†
Type 4: Low ANC 4+ / Low FBD	10	Below median	Below median	Badghis, Farah†, Ghor, Helmand, Kandahar†, Nuristan, Paktya†, Samangan†, Uruzgan†, Zabul

Thresholds are the medians across the 34 province-specific survey-weighted estimates: ANC 4+ coverage = 40.4% and facility-based delivery = 77.5%. These are not national population-weighted prevalence estimates; classification used unrounded values. † indicates that the 95% CI for ANC 4+ and/or facility-based delivery crossed the corresponding median threshold. Exact bootstrap retention probabilities are reported in Supplementary Table S1.

Quadrant assignment was not equally stable across provinces. Provinces far from both medians generally retained their point-estimate classification across bootstrap replicates, whereas provinces close to one or both thresholds frequently moved between adjacent quadrants. The lowest retention probabilities were observed for Maidan Wardak and Takhar (approximately 40%), Baghlan (41%), Panjshir (47%), Kunduz (50%), Paktya (52%), Paktika and Samangan (54%), Jawzjan (55%), and Faryab (56%). These results support interpreting quadrant membership as a descriptive point estimate rather than as a fixed provincial characteristic. Figure 1 shows the geographic distribution, Figure 2 displays the two indicators with confidence intervals and median lines, and Supplementary Table S1 provides exact uncertainty estimates.

**Figure 1.**
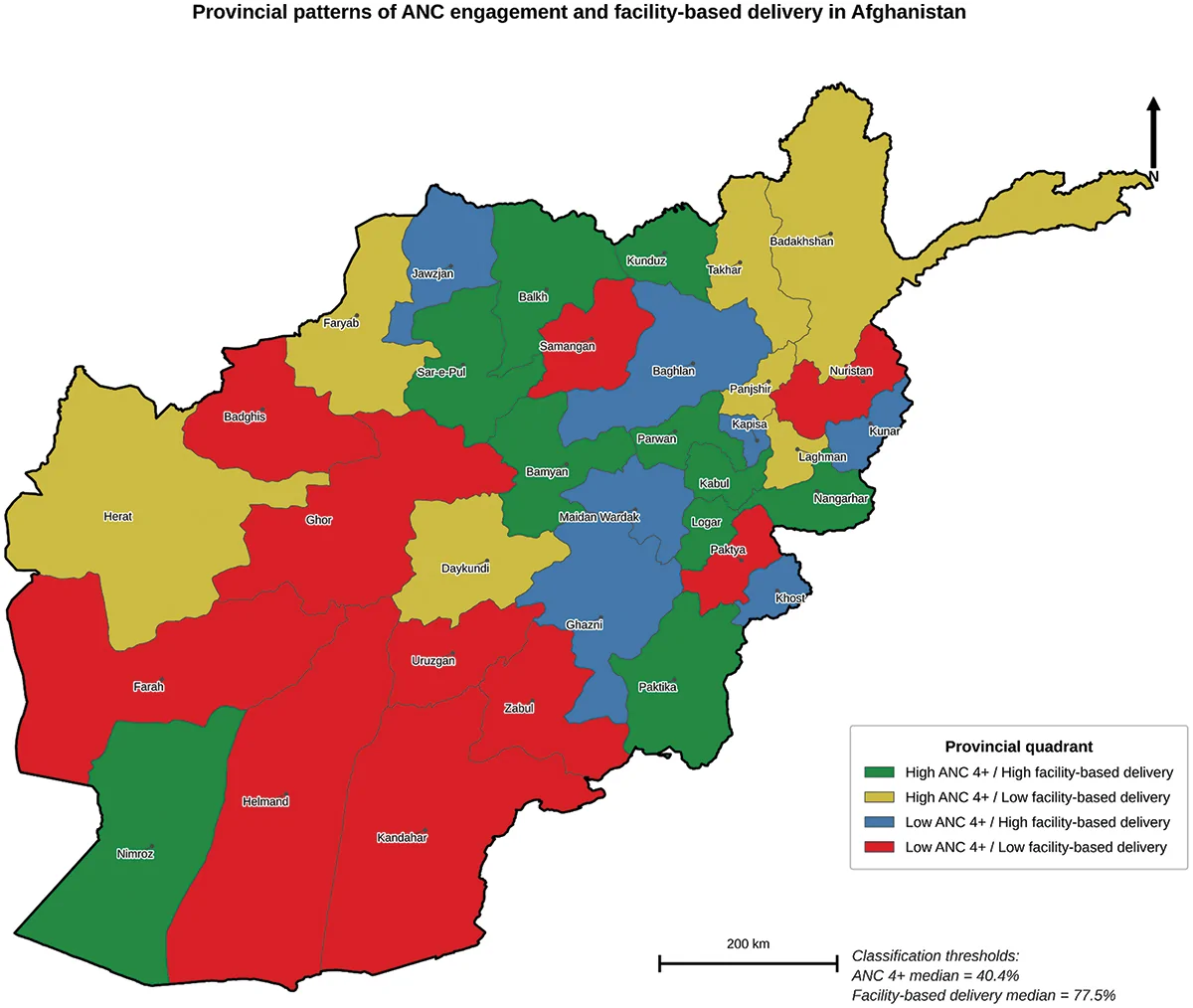
Median-based provincial quadrants of antenatal care (ANC) engagement and facility-based delivery among ANC-attending women, Afghanistan MICS 2022–23. Provinces are classified according to whether survey-weighted ANC 4+ coverage and facility-based delivery fall above or below the medians across the 34 province-specific estimates (ANC 4+: 40.4%; facility-based delivery: 77.5%). The four descriptive quadrants are high ANC 4+/high facility-based delivery, high ANC 4+/low facility-based delivery, low ANC 4+/high facility-based delivery, and low ANC 4+/low facility-based delivery. Thresholds are descriptive provincial medians and are not national population-weighted prevalence estimates.

**Figure 2.**
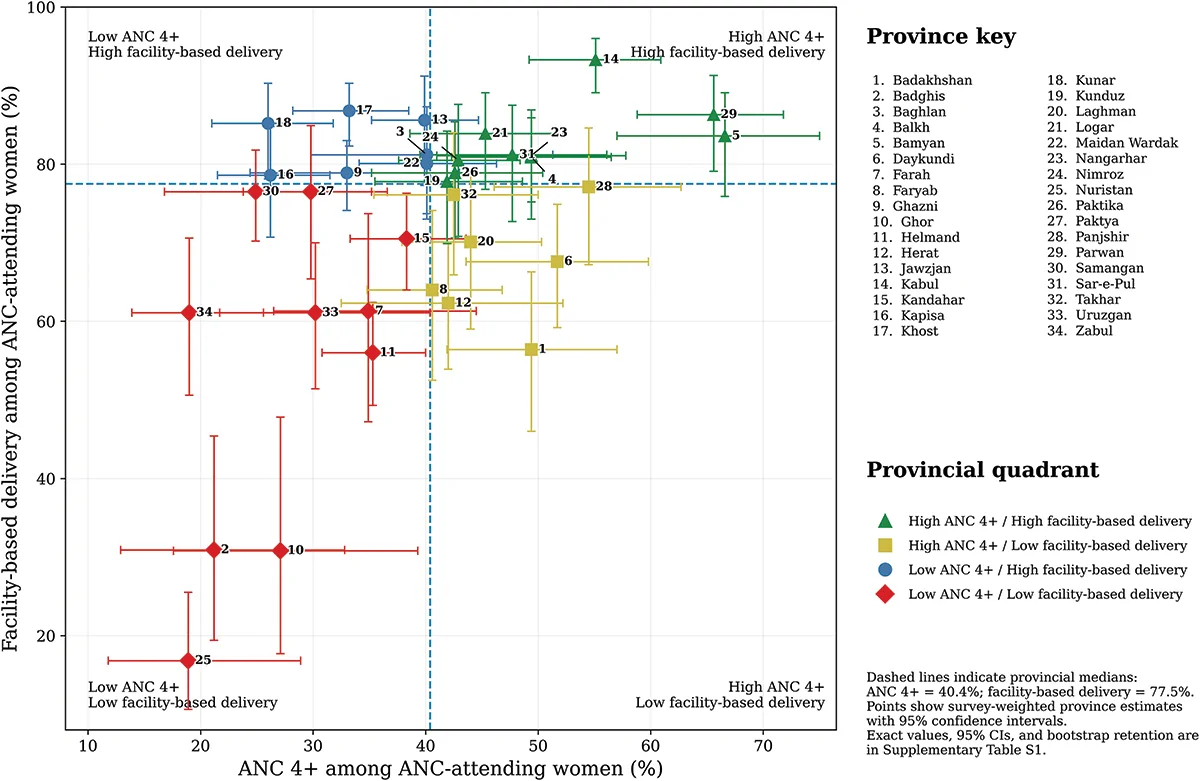
Province-level ANC 4+ coverage versus facility-based delivery among ANC-attending women, Afghanistan MICS 2022–23. Vertical and horizontal lines show the medians across the 34 province-specific survey-weighted estimates (ANC 4+: 40.4%; facility-based delivery: 77.5%). Error bars show 95% confidence intervals for both indicators. Provinces close to one or both thresholds show greater classification uncertainty; exact bootstrap quadrant-retention probabilities are reported in Supplementary Table S1.

### Sensitivity analysis by pregnancy-risk proxy

The interaction between ≥ 8 ANC contacts and the higher-risk proxy was negative (aOR 0.43; 95% CI 0.25 to 0.75; *p* = 0.003), indicating that the association with ≥8 contacts was weaker among women meeting the proxy. Adjusted probabilities of facility-based delivery were 72.5% for lower-risk women with 1–3 contacts and 87.8% for lower-risk women with ≥8 contacts, compared with 71.0% and 75.6%, respectively, among women meeting the higher-risk proxy. The pattern therefore demonstrates effect modification by the study’s age/parity proxy but does not establish the direction of causation or exclude residual obstetric confounding.

### Skilled birth attendance as an alternative outcome

The weighted skilled birth attendance rate was 75.4%, compared with 74.5% for facility-based delivery, an absolute difference of approximately 1.0% point. Main adjusted associations remained similar under the two outcome definitions: the adjusted odds ratio for 4–7 versus 1–3 contacts was 1.49 for facility-based delivery and 1.40 for skilled birth attendance, while the corresponding estimates for ≥8 contacts were 1.93 and 1.85. Twenty-eight of 34 provinces retained the same median-based quadrant when skilled birth attendance replaced facility-based delivery; Panjshir, Baghlan, Paktya, Takhar, Kunduz, and Balkh shifted between adjacent quadrants (Supplementary Table S2). This sensitivity analysis indicates that the principal individual-level patterns were not dependent on defining the delivery endpoint solely by place of birth.

## Discussion

This study examines continuity after entry into antenatal care rather than initiation of maternal healthcare. Among ever-married women with a recent live birth who had attended at least one ANC contact, 25.5% subsequently delivered outside a health facility. This finding indicates that a substantial gap remains even among women who have already engaged with the health system during pregnancy. The analysis further shows that higher ANC contact frequency was associated with greater facility-based delivery after adjustment for socioeconomic, demographic, timing, and measured clinical-content variables, while the three-item content measure showed a smaller and non-monotonic association. At the same time, province-level analyses demonstrated pronounced heterogeneity in both ANC 4+ coverage and facility-based delivery and showed that several median-based quadrant assignments were uncertain rather than fixed.

A central contribution of this analysis is placing ANC frequency and measured clinical content on a common adjusted probability scale. The ≥ 8 versus 1–3 contact contrast was associated with a 10.3%-point higher adjusted probability of facility-based delivery, whereas the contrast between content scores 3 and 0 was 4.6 points. These estimates are not a formal ranking of causal importance, but they are more directly comparable than odds ratios defined on different exposure scales. The results also show overlap between the two dimensions: mean measured content increased with contact frequency, and both associations attenuated after mutual adjustment.

That overlap is substantively plausible. Repeated contacts may create additional opportunities for clinical monitoring, counselling, birth preparedness, recognition of complications, referral, and familiarity with providers and facilities [13,24,25]. Measured content may therefore be one pathway through which repeated contact is associated with subsequent care-seeking, while frequency may also capture broader engagement not represented by the three clinical items. The absence of a clear frequency-by-content interaction does not imply that the two are unrelated; rather, the data did not show strong evidence that the association of one varied across categories of the other. Formal mediation inference was not supported by the available temporal information.

The three-item measured clinical-content score should consequently be interpreted narrowly. Blood pressure measurement, urine testing, and blood testing are important components of ANC, but they do not capture counselling, respectful care, communication, continuity, referral quality, patient experience, or facility readiness [10,11]. The score was also non-monotonic across intermediate categories, and 38.7% of women were already at the maximum value, creating a ceiling. Because the timing and indication of these services differ across pregnancy, the score is useful as a measure of selected clinical content but not as a comprehensive quality index [14].

The conditional non-facility-delivery rate observed here is consistent with a broader literature showing losses between ANC and childbirth care in low- and middle-income settings. Multicountry analyses and country-specific studies have reported substantial discontinuity after ANC booking or contact, including in sub-Saharan Africa, Ethiopia, and Pakistan [26–28]. Direct numerical comparison, however, is difficult because studies differ in the stage used to define entry into the continuum, whether adequate ANC rather than any ANC is required, and whether the endpoint is facility-based delivery or skilled attendance. The Afghanistan estimate is therefore best interpreted as a conditional measure among ANC-attending women rather than as evidence that the country performs better or worse than pooled estimates from other settings.

The provincial results add a second level of interpretation. Facility-based delivery among ANC-attending women ranged from 16.8% in Nuristan to 93.3% in Kabul, showing that the national conditional estimate conceals very large subnational differences. The moderate ecological correlation between ANC 4+ coverage and facility-based delivery indicates that provinces with deeper ANC engagement tended, on average, also to have greater facility-based delivery. In contrast, province-level correlations with the three measured clinical components were weak. These ecological patterns complement the individual-level results but should not be treated as evidence that the same mechanisms operate at both levels.

The high ANC 4+/low facility-based-delivery quadrant is particularly informative descriptively because it identifies provinces in which relatively deeper ANC engagement coexists with below-median facility-based delivery. In this classification, this group includes Laghman, Panjshir, Badakhshan, Takhar, Daykundi, Faryab, and Herat. These provinces are geographically and socioeconomically heterogeneous, so their shared quadrant should not be interpreted as proof of a single barrier such as terrain or transport. Their common feature is simply the observed combination of the two indicators. Potential explanations could include transport constraints during labour, referral pathways, household costs, facility readiness, insecurity, decision-making dynamics, or sociocultural factors, but distinguishing among these mechanisms requires province-specific data [17,18,29,30].

The low ANC 4+/low facility-based-delivery quadrant describes a different pattern: both indicators fall below their respective provincial medians. Provinces such as Nuristan, Ghor, and Badghis also lie far below the facility-delivery median, suggesting substantial deficits in childbirth care among women who did reach ANC. Previous Afghan studies have documented marked geographic inequalities in service access and travel time, and conflict and health-system fragility may further constrain both routine pregnancy care and access during labour [16–18,31]. More broadly, health-systems research emphasises that nominal service contact does not by itself establish that care is accessible, effective, or high quality [32]. Nevertheless, the quadrant itself cannot establish whether limited service supply, physical access, household demand, perceived quality, or another factor is the dominant explanation.

Conversely, the low ANC 4+/high facility-based-delivery quadrant includes provinces where facility-based delivery is relatively high despite lower ANC 4+ coverage. This pattern may reflect differences in how pregnancy and childbirth services are accessed or organised across provinces. Provinces in the high/high quadrant, by contrast, have above-median levels of both indicators. Together, the four quadrants provide a descriptive summary of how ANC engagement and facility-based delivery vary across Afghanistan.

The bootstrap analysis is important for interpreting these provincial patterns. Several provinces lie close to one or both median thresholds and changed quadrants frequently under resampling. Maidan Wardak, Takhar, Baghlan, Panjshir, Kunduz, Paktya, Paktika, Samangan, Jawzjan, and Faryab had particularly low point-estimate retention. For these provinces, attaching substantive meaning to a single quadrant label would overstate precision. The confidence intervals and retention probabilities are more informative than the label alone. Provinces that are far from the thresholds can be described with greater confidence, but even there the estimates remain descriptive measures of service use rather than direct diagnoses of health-system performance.

Socioeconomic inequalities remained prominent after adjustment. Women in the richest wealth quintile had substantially higher odds of facility-based delivery than those in the poorest quintile, and the crude wealth gradient was even larger. Rural residence was associated with markedly lower facility-based delivery despite adjustment for wealth and education. These findings are consistent with previous Afghan evidence showing that financial resources, geography, and physical accessibility remain central to maternal-health service use [3,9,16–18]. They also underscore that continuity after ANC initiation cannot be understood solely through what occurs inside ANC visits; the ability to reach and use childbirth care remains strongly structured by household and place-level conditions.

Education showed a graded association at higher levels, whereas primary education alone was not statistically significant after adjustment. This pattern should be interpreted cautiously rather than as evidence that primary schooling has no value for maternal health. Education is correlated with wealth, residence, autonomy, health literacy, and many other factors, and the adjusted coefficient represents the association remaining after those measured covariates are taken into account. Higher parity was inversely associated with facility-based delivery, which may reflect prior birth experience, competing household demands, or unmeasured preferences, although these mechanisms are not directly measured [3,9,33].

Maternal age deserves separate consideration because its adjusted association was positive even though the crude association was close to null. Once parity and the other covariates were held constant, a 10-year increase in maternal age was associated with higher odds of facility-based delivery. Maternal age and parity are closely related, so the adjusted coefficient should be read as the conditional association of age at a given parity rather than as a simple population age gradient. Possible explanations include greater perceived obstetric risk, accumulated experience with formal healthcare, or residual differences in household circumstances, but the analysis cannot distinguish among them.

The conditional sample frame is both a strength and an important interpretive boundary. By focusing on women who had already initiated ANC, the analysis separates non-continuation from non-initiation and highlights a group that is often merged into broader facility-delivery analyses. This perspective is particularly useful for programme planning because it asks what happens after women have already made contact with maternal-health services. At the same time, conditioning on ANC initiation selects a subgroup of women whose access, preferences, resources, and care-seeking propensity may differ from those who never entered ANC. The resulting associations therefore should not be generalised as the effect of ANC use in the full population.

The pregnancy-risk sensitivity analysis showed that the association between ≥8 ANC contacts and facility-based delivery was weaker among women meeting the age/parity-based higher-risk proxy. This suggests that the relationship between ANC contact frequency and facility-based delivery may differ according to maternal risk profile. However, the proxy does not directly capture pregnancy complications or clinical severity, and residual confounding by factors related to care-seeking and access cannot be excluded.

The skilled-birth-attendance sensitivity analysis provides a useful robustness check on the choice of delivery outcome. Skilled attendance was approximately 1.0% point higher than facility-based delivery because a minority of non-facility births involved a skilled provider. The adjusted associations with ANC frequency remained very similar under the alternative definition, and most provinces retained the same quadrant. These findings suggest that the main individual-level patterns are not an artefact of using place of delivery rather than skilled attendance, while also reinforcing that the two outcomes are conceptually distinct and should not be treated as interchangeable.

From a programme perspective, the findings support closer attention to continuity after ANC initiation without implying that increasing contacts alone will increase facility-based delivery. Repeated engagement creates opportunities for counselling, birth planning, referral, and monitoring, yet women able to accumulate more contacts may also face fewer structural barriers to facility childbirth. Programmes should therefore consider sustained engagement during pregnancy alongside transport, referral, affordability, service readiness, and community support, with priorities tailored to local settings [15,18,25,29,30].

The provincial analysis may help identify where further investigation is most needed. In provinces with high ANC 4+ coverage but lower facility-based delivery, local analyses could examine referral completion, labour-time travel barriers, costs, perceived quality, and childbirth-service readiness. Where both indicators are low, service availability and initial access may also be important; where facility-based delivery is relatively high despite lower ANC depth, continuity earlier in pregnancy may warrant attention. These remain hypotheses generated by observed patterns rather than conclusions established by quadrant labels.

### Strengths and limitations

This study has several strengths. It uses a large national household survey with representation across all 34 provinces and accounts for clustering and sampling weights in the main regression analysis. The conditional analytical frame isolates continuity after ANC initiation, while categorical modelling and standardised probability differences improve comparison of ANC frequency and measured content. The provincial analysis also incorporates confidence intervals and bootstrap classification-retention probabilities rather than relying only on point-estimate quadrants.

Several limitations should be considered. First, the cross-sectional observational design precludes causal inference. ANC contact frequency and facility-based delivery may share unmeasured determinants, including geographic accessibility, transport, household resources, decision-making, prior healthcare experiences, perceived need, and care-seeking propensity. The observed associations, therefore, should not be interpreted as effects that would necessarily result from increasing ANC contacts.

Second, the analysis is conditional on having attended at least one ANC contact. This restriction is intentional, but it may introduce selection bias if unmeasured factors influence both ANC initiation and subsequent facility-based delivery. The findings therefore apply specifically to ANC-attending ever-married women with a recent live birth and should not be extrapolated as causal estimates for all women with a recent birth.

Third, the MICS does not directly measure pregnancy complications or clinical severity. The higher-risk proxy based on maternal age and parity is therefore incomplete. The interaction analysis can show that the frequency association differed according to this proxy, but it cannot exclude residual confounding by obstetric risk or establish the temporal direction of the association.

Fourth, the measured clinical-content score has interpretive limitations. Its components differ in timing and indication, are self-reported, and omit counselling, respectful care, communication, provider competence, continuity, and facility readiness. The score also has a ceiling and showed a non-monotonic relationship with facility-based delivery. It should therefore be interpreted as a limited indicator of selected ANC services rather than comprehensive care quality.

Fifth, the provincial quadrants are based on medians across 34 province-specific estimates. These medians are methodological reference points, not externally validated performance standards, and provinces close to either threshold can change category because of sampling variability. Province-level sample sizes also vary, affecting precision. For these reasons, confidence intervals and bootstrap retention probabilities should be considered alongside quadrant membership, and the classifications should be used descriptively rather than as fixed labels.

Sixth, the MICS maternal-health questionnaire is administered to ever-married women aged 15–49 years, so the findings are not directly generalisable to never-married women. The two-year live-birth reference period also spans pregnancies occurring before and after the August 2021 political transition, meaning the study predominantly, but not exclusively, reflects post-transition maternal care. A small number of survey PSUs were inaccessible, and self-reported delivery and ANC information may be affected by recall or social-desirability bias.

Finally, facility-based delivery and skilled birth attendance are related but not identical outcomes. The skilled-attendance analysis showed similar main associations, which provides reassurance about the robustness of the findings to outcome definition, but it does not remove the broader limitations of the observational design or the conditional sample.

## Conclusions

Among ever-married women with a recent live birth in Afghanistan who had already initiated antenatal care, approximately one in four did not subsequently deliver in a health facility. Higher ANC contact frequency was consistently associated with greater facility-based delivery, and when frequency and measured clinical content were expressed on the same adjusted probability scale, the largest contrast was observed across ANC-frequency categories. The three-item content measure showed a smaller, non-monotonic association and should be interpreted as selected clinical content rather than comprehensive ANC quality.

The provincial analysis further demonstrates that continuity after ANC initiation is highly heterogeneous across Afghanistan. Median-based quadrants provide a useful descriptive summary of how ANC 4+ coverage and facility-based delivery coexist across provinces, but they do not define fixed health-system types and should be interpreted alongside province-specific confidence intervals and bootstrap classification stability. Several provinces close to the thresholds had substantial classification uncertainty, whereas provinces far from the thresholds showed clearer differences in service-use patterns.

These findings support greater attention to continuity between pregnancy care and childbirth. Higher ANC contact frequency was associated with greater facility-based delivery, while broader barriers related to transport, referral, affordability, service readiness, and household decision-making are also likely to shape whether women reach facilities for childbirth. Maternal-health programmes should therefore combine efforts to sustain engagement during pregnancy with strategies that address barriers to delivery care, informed by province-specific patterns of service use.

## Supplementary Material

STROBE_Checklist_Revised.docx

Supplementary_Material_GHA_FINAL.docx

## Data Availability

The Afghanistan Multiple Indicator Cluster Survey 2022–23 microdata are publicly available through UNICEF’s MICS programme at https://mics.unicef.org/country-profiles/afghanistan/3746. Access is subject to the programme’s standard data-use request process. Analysis code is available from the corresponding author on reasonable request.
