# Supplementary material for: From antenatal contact to facility-based delivery: a provincial analysis of dropout along the maternal continuum of care in Afghanistan using the 2022–23 MICS
Source: Glob Health Action. 2026 Oct 20;19(1):2750043. doi: 10.1080/16549716.2026.2750043 (PMC13686201; doi:10.1080/16549716.2026.2750043)
Supplement: Supplementary file 1 [file ZGHA_A_2750043_SM4600.docx]

**Supplementary Table S1. Province-specific facility-based delivery, ANC 4+ coverage, uncertainty, and median-based quadrant classification among ANC-attending women with a recent live birth, Afghanistan MICS 2022–23**

Notes: Provinces are sorted by facility-based delivery (ascending). Estimates are survey-weighted. The median across the 34 province-specific estimates was 77.5% for facility-based delivery and 40.4% for ANC 4+ coverage; unrounded medians were used for classification. Ninety-five percent confidence intervals are logit-scale intervals around the survey-weighted province estimates. Variances for the survey-weighted provincial proportions were estimated by Taylor linearisation, treating primary sampling units as clusters within the MICS sampling strata; the resulting standard errors were transformed to the logit scale for interval construction, and the confidence limits were back-transformed to probabilities. Bootstrap retention is the percentage of resamples in which the province retained its point-estimate quadrant. “Threshold overlap” indicates whether the 95% confidence interval crossed the corresponding provincial median. ANC-attending n is the unweighted cohort count; indicator-specific denominators may be slightly smaller because of item nonresponse.

*Quadrants: Type 1 = high ANC 4+ / high facility-based delivery; Type 2 = high ANC 4+ / low facility-based delivery; Type 3 = low ANC 4+ / high facility-based delivery; Type 4 = low ANC 4+ / low facility-based delivery. These are descriptive median-based quadrants, not fixed health-system types.*

| **Rank** | **Province** | **ANC-attending n** | **Facility-based delivery % (95% CI)** | **Non-facility delivery %** | **ANC 4+ % (95% CI)** | **Point-estimate quadrant** | **Bootstrap retention %** | **Threshold overlap** |
| --- | --- | --- | --- | --- | --- | --- | --- | --- |
| 1 | Nuristan | 161 | **16.8 (10.6–25.5)** | 83.2 | 18.9 (11.8–28.9) | Type 4: Low ANC 4+ / Low FBD | 100.0 | Neither |
| 2 | Ghor | 88 | **30.8 (17.7–47.8)** | 69.2 | 27.1 (17.6–39.3) | Type 4: Low ANC 4+ / Low FBD | 98.8 | Neither |
| 3 | Badghis | 120 | **30.9 (19.4–45.4)** | 69.1 | 21.2 (12.9–32.8) | Type 4: Low ANC 4+ / Low FBD | 100.0 | Neither |
| 4 | Helmand | 379 | **56.0 (49.3–62.4)** | 44.0 | 35.3 (30.8–40.0) | Type 4: Low ANC 4+ / Low FBD | 98.2 | Neither |
| 5 | Badakhshan | 216 | **56.4 (46.0–66.3)** | 43.6 | 49.4 (41.9–57.0) | Type 2: High ANC 4+ / Low FBD | 99.3 | Neither |
| 6 | Zabul | 297 | **61.1 (50.6–70.6)** | 38.9 | 19.0 (13.9–25.6) | Type 4: Low ANC 4+ / Low FBD | 100.0 | Neither |
| 7 | Uruzgan | 102 | **61.1 (51.4–70.0)** | 38.9 | 30.2 (21.7–40.4) | Type 4: Low ANC 4+ / Low FBD | 99.3 | ANC 4+ |
| 8 | Farah | 258 | **61.3 (47.2–73.7)** | 38.7 | 34.9 (26.5–44.5) | Type 4: Low ANC 4+ / Low FBD | 88.8 | ANC 4+ |
| 9 | Herat | 303 | **62.3 (53.9–69.9)** | 37.7 | 42.0 (32.5–52.2) | Type 2: High ANC 4+ / Low FBD | 61.3 | ANC 4+ |
| 10 | Faryab | 299 | **64.0 (52.5–74.1)** | 36.0 | 40.6 (34.8–46.8) | Type 2: High ANC 4+ / Low FBD | 56.3 | ANC 4+ |
| 11 | Daykundi | 251 | **67.6 (59.2–74.9)** | 32.4 | 51.7 (43.6–59.8) | Type 2: High ANC 4+ / Low FBD | 99.1 | Neither |
| 12 | Laghman | 297 | **70.1 (59.0–79.2)** | 29.9 | 44.0 (37.9–50.3) | Type 2: High ANC 4+ / Low FBD | 84.9 | Both |
| 13 | Kandahar | 390 | **70.5 (64.0–76.3)** | 29.5 | 38.3 (33.3–43.6) | Type 4: Low ANC 4+ / Low FBD | 72.9 | ANC 4+ |
| 14 | Takhar | 289 | **76.1 (65.9–84.0)** | 23.9 | 42.5 (35.4–50.0) | Type 2: High ANC 4+ / Low FBD | 40.5 | Both |
| 15 | Paktya | 331 | **76.5 (65.4–84.9)** | 23.5 | 29.8 (23.8–36.6) | Type 4: Low ANC 4+ / Low FBD | 51.8 | FBD |
| 16 | Samangan | 174 | **76.5 (70.2–81.8)** | 23.5 | 24.9 (16.8–35.2) | Type 4: Low ANC 4+ / Low FBD | 54.1 | FBD |
| 17 | Panjshir | 203 | **77.1 (67.2–84.6)** | 22.9 | 54.5 (46.1–62.7) | Type 2: High ANC 4+ / Low FBD | 47.2 | FBD |
| 18 | Kunduz | 327 | **77.8 (69.9–84.2)** | 22.2 | 41.9 (35.5–48.6) | Type 1: High ANC 4+ / High FBD | 50.4 | Both |
| 19 | Kapisa | 229 | **78.6 (70.7–84.8)** | 21.4 | 26.2 (21.5–31.5) | Type 3: Low ANC 4+ / High FBD | 70.1 | FBD |
| 20 | Ghazni | 261 | **78.9 (74.1–83.0)** | 21.1 | 33.0 (24.4–43.0) | Type 3: Low ANC 4+ / High FBD | 73.9 | Both |
| 21 | Paktika | 317 | **78.9 (70.6–85.4)** | 21.1 | 42.6 (35.2–50.4) | Type 1: High ANC 4+ / High FBD | 54.1 | Both |
| 22 | Maidan Wardak | 255 | **80.1 (73.7–85.3)** | 19.9 | 40.1 (34.1–46.3) | Type 3: Low ANC 4+ / High FBD | 40.3 | Both |
| 23 | Nimroz | 309 | **80.5 (70.8–87.6)** | 19.5 | 42.9 (37.6–48.4) | Type 1: High ANC 4+ / High FBD | 72.3 | Both |
| 24 | Balkh | 266 | **80.9 (73.0–86.9)** | 19.1 | 49.4 (42.3–56.5) | Type 1: High ANC 4+ / High FBD | 87.4 | FBD |
| 25 | Nangarhar | 344 | **81.1 (75.2–85.9)** | 18.9 | 49.4 (41.0–57.8) | Type 1: High ANC 4+ / High FBD | 89.7 | FBD |
| 26 | Baghlan | 208 | **81.2 (73.0–87.3)** | 18.8 | 40.1 (29.8–51.3) | Type 3: Low ANC 4+ / High FBD | 40.9 | Both |
| 27 | Sar-e-Pul | 338 | **81.2 (72.7–87.5)** | 18.8 | 47.7 (39.5–56.1) | Type 1: High ANC 4+ / High FBD | 86.9 | Both |
| 28 | Bamyan | 256 | **83.6 (75.9–89.1)** | 16.4 | 66.6 (57.0–75.0) | Type 1: High ANC 4+ / High FBD | 97.3 | FBD |
| 29 | Logar | 376 | **83.9 (76.8–89.1)** | 16.1 | 45.3 (38.6–52.2) | Type 1: High ANC 4+ / High FBD | 94.0 | Both |
| 30 | Kunar | 243 | **85.2 (78.1–90.3)** | 14.8 | 26.0 (21.0–31.8) | Type 3: Low ANC 4+ / High FBD | 99.2 | Neither |
| 31 | Jawzjan | 306 | **85.6 (77.3–91.2)** | 14.4 | 39.9 (35.2–44.7) | Type 3: Low ANC 4+ / High FBD | 55.2 | Both |
| 32 | Parwan | 327 | **86.3 (79.1–91.3)** | 13.7 | 65.6 (58.8–71.8) | Type 1: High ANC 4+ / High FBD | 99.6 | Neither |
| 33 | Khost | 457 | **86.8 (82.3–90.3)** | 13.2 | 33.2 (28.2–38.5) | Type 3: Low ANC 4+ / High FBD | 99.5 | Neither |
| 34 | Kabul | 291 | **93.3 (89.1–96.0)** | 6.7 | 55.1 (49.2–60.9) | Type 1: High ANC 4+ / High FBD | 100.0 | Neither |

**Supplementary Table S2. Sensitivity analysis: adjusted associations with facility-based delivery (FBD) versus skilled birth attendance (SBA) among ANC-attending women, Afghanistan MICS 2022–23 (complete-case n = 9,223)**

FBD = delivery in a health facility. SBA = delivery attended by a medical doctor or nurse/midwife, consistent with Afghanistan MICS indicator TM.9. Both models include the same covariates simultaneously and use the same complete-case sample. Measured ANC clinical content is modelled categorically (scores 0–3), consistent with the primary analysis. Estimates are survey-weighted with PSU-clustered robust standard errors.

| **Variable** | **Category (ref)** | **aOR (95% CI) — FBD** | **p** | **aOR (95% CI) — SBA** | **p** |
| --- | --- | --- | --- | --- | --- |
| **Wealth quintile** | Poorest (ref) | 1.00 | — | 1.00 | — |
|  | Second quintile | 1.32 (1.09–1.60) | 0.005 | 1.32 (1.08–1.61) | 0.006 |
|  | Middle quintile | 1.84 (1.48–2.28) | <0.001 | 1.81 (1.47–2.21) | <0.001 |
|  | Fourth quintile | 2.65 (2.11–3.32) | <0.001 | 2.71 (2.12–3.48) | <0.001 |
|  | Richest quintile | 4.88 (3.51–6.78) | <0.001 | 5.46 (3.92–7.61) | <0.001 |
| **Education** | No education (ref) | 1.00 | — | 1.00 | — |
|  | Primary school | 1.25 (0.97–1.62) | 0.087 | 1.39 (1.07–1.80) | 0.013 |
|  | Lower secondary | 1.58 (1.09–2.28) | 0.015 | 1.58 (1.13–2.21) | 0.008 |
|  | Upper secondary | 1.80 (1.22–2.65) | 0.003 | 1.82 (1.24–2.68) | 0.002 |
|  | Higher / university | 4.88 (2.13–11.17) | <0.001 | 3.21 (1.56–6.60) | 0.001 |
| **Residence** | Urban (ref) | 1.00 | — | 1.00 | — |
|  | Rural | 0.50 (0.35–0.71) | <0.001 | 0.56 (0.41–0.76) | <0.001 |
| **Maternal age** | Per 10 years | 1.46 (1.22–1.73) | <0.001 | 1.49 (1.25–1.79) | <0.001 |
| **Parity** | Per additional child | 0.90 (0.87–0.94) | <0.001 | 0.92 (0.88–0.95) | <0.001 |
| **ANC frequency** | 1–3 visits (ref) | 1.00 | — | 1.00 | — |
|  | 4–7 visits | 1.49 (1.25–1.77) | <0.001 | 1.40 (1.18–1.66) | <0.001 |
|  | 8+ visits | 1.93 (1.45–2.57) | <0.001 | 1.85 (1.38–2.48) | <0.001 |
| **First-trimester ANC** | No (ref) | 1.00 | — | 1.00 | — |
|  | Yes | 1.23 (1.05–1.45) | 0.011 | 1.21 (1.03–1.42) | 0.019 |
| **Measured ANC clinical content** | Score 0 (ref) | 1.00 | — | 1.00 | — |
|  | Score 1 | 0.87 (0.72–1.06) | 0.176 | 0.96 (0.79–1.16) | 0.650 |
|  | Score 2 | 1.02 (0.81–1.28) | 0.859 | 1.12 (0.90–1.39) | 0.316 |
|  | Score 3 | 1.33 (1.06–1.66) | 0.012 | 1.36 (1.09–1.68) | 0.006 |

**Supplementary Table S2 (continued). Provincial median-based quadrant classification under FBD versus SBA outcomes (n = 34 provinces**)

Provincial median thresholds: FBD = 77.5%, SBA = 78.0%, ANC 4+ = 40.4%. Rows shaded pale yellow changed quadrant when SBA replaced FBD.

Six provinces changed between adjacent quadrants: Panjshir, Baghlan, Paktya, Takhar, Kunduz, and Balkh. The remaining 28 provinces retained their FBD-based quadrant. Quadrant counts remained 10/7/7/10 under both outcomes. Provincial ANC 4+ coverage was correlated with SBA at r = 0.52 (p = 0.001).

*Quadrants: Type 1 = high ANC 4+ / high delivery outcome; Type 2 = high ANC 4+ / low delivery outcome; Type 3 = low ANC 4+ / high delivery outcome; Type 4 = low ANC 4+ / low delivery outcome.*

| **Province** | **FBD %** | **SBA %** | **FBD quadrant** | **SBA quadrant** |
| --- | --- | --- | --- | --- |
| Nuristan | 16.8 | 18.8 | Type 4 | Type 4 |
| Ghor | 30.8 | 39.5 | Type 4 | Type 4 |
| Badghis | 30.9 | 34.4 | Type 4 | Type 4 |
| Helmand | 56.0 | 56.8 | Type 4 | Type 4 |
| Badakhshan | 56.4 | 59.8 | Type 2 | Type 2 |
| Zabul | 61.1 | 60.6 | Type 4 | Type 4 |
| Uruzgan | 61.1 | 64.3 | Type 4 | Type 4 |
| Farah | 61.3 | 61.1 | Type 4 | Type 4 |
| Herat | 62.3 | 66.6 | Type 2 | Type 2 |
| Faryab | 64.0 | 65.3 | Type 2 | Type 2 |
| Daykundi | 67.6 | 70.9 | Type 2 | Type 2 |
| Laghman | 70.1 | 71.8 | Type 2 | Type 2 |
| Kandahar | 70.5 | 71.1 | Type 4 | Type 4 |
| Takhar | 76.1 | 79.1 | Type 2 | Type 1 |
| Paktya | 76.5 | 82.3 | Type 4 | Type 3 |
| Samangan | 76.5 | 77.0 | Type 4 | Type 4 |
| Panjshir | 77.1 | 80.2 | Type 2 | Type 1 |
| Kunduz | 77.8 | 72.4 | Type 1 | Type 2 |
| Kapisa | 78.6 | 83.4 | Type 3 | Type 3 |
| Ghazni | 78.9 | 79.4 | Type 3 | Type 3 |
| Paktika | 78.9 | 83.2 | Type 1 | Type 1 |
| Maidan Wardak | 80.1 | 80.9 | Type 3 | Type 3 |
| Nimroz | 80.5 | 82.0 | Type 1 | Type 1 |
| Balkh | 80.9 | 74.8 | Type 1 | Type 2 |
| Nangarhar | 81.1 | 82.8 | Type 1 | Type 1 |
| Baghlan | 81.2 | 69.8 | Type 3 | Type 4 |
| Sar-e-Pul | 81.2 | 83.3 | Type 1 | Type 1 |
| Bamyan | 83.6 | 83.7 | Type 1 | Type 1 |
| Logar | 83.9 | 84.3 | Type 1 | Type 1 |
| Kunar | 85.2 | 87.3 | Type 3 | Type 3 |
| Jawzjan | 85.6 | 88.5 | Type 3 | Type 3 |
| Parwan | 86.3 | 88.0 | Type 1 | Type 1 |
| Khost | 86.8 | 88.1 | Type 3 | Type 3 |
| Kabul | 93.3 | 95.4 | Type 1 | Type 1 |

**Supplementary Table S3. Distribution of measured ANC clinical-content scores and conditional facility-based delivery among ANC-attending women, Afghanistan MICS 2022–23**

*Panel A: score distribution and corresponding weighted facility-based delivery. Panel B: component combinations underlying each score. Panel C: measured content by ANC contact frequency. Denominators vary slightly across panels according to non-missing information required for each analysis. All percentages are survey-weighted; n values are unweighted.*

**Panel A. Score distribution and conditional facility-based delivery**

| **Measured ANC content score (0–3)** | **Raw n** | **Weighted %** | **Facility-based delivery (weighted %)** |
| --- | --- | --- | --- |
| 0 | 1,173 | 11.7% | 70.9% |
| 1 | 2,686 | 29.3% | 66.0% |
| 2 | 1,951 | 20.4% | 73.2% |
| 3 | 3,438 | 38.7% | 82.6% |

**Panel B. Component combinations underlying each score level**

| **BP** | **Urine** | **Blood** | **Score** | **Raw n** | **Weighted %** | **FBD (weighted %)** |
| --- | --- | --- | --- | --- | --- | --- |
| No | No | No | 0 | 1,173 | 11.7% | 70.9% |
| No | No | Yes | 1 | 186 | 1.6% | 79.2% |
| No | Yes | No | 1 | 287 | 2.1% | 71.5% |
| No | Yes | Yes | 2 | 300 | 2.1% | 79.5% |
| Yes | No | No | 1 | 2,213 | 25.6% | 64.7% |
| Yes | No | Yes | 2 | 930 | 10.7% | 73.1% |
| Yes | Yes | No | 2 | 721 | 7.6% | 71.6% |
| Yes | Yes | Yes | 3 | 3,438 | 38.7% | 82.6% |

**Panel C. Measured clinical content by ANC contact frequency**

| **ANC contact frequency** | **n** | **Mean content score** | **BP (%)** | **Urine (%)** | **Blood (%)** |
| --- | --- | --- | --- | --- | --- |
| 1-3 contacts | 5,486 | 1.62 | 79.2 | 40.6 | 42.2 |
| 4-7 contacts | 2,966 | 2.12 | 85.8 | 61.3 | 65.1 |
| 8+ contacts | 797 | 2.41 | 92.1 | 71.9 | 77.1 |

*BP = blood pressure measurement; Urine = urine sample taken; Blood = blood sample taken. The unadjusted relationship between the score and facility-based delivery is non-monotonic: score 0 had 70.9% facility-based delivery, compared with 66.0% for score 1. The score-1 group is dominated by receipt of blood pressure measurement alone (25.6% of women with complete score/outcome data; facility-based delivery 64.7%). A ceiling is also evident, with 38.7% receiving all three measured components. Mean measured content increased from 1.62 among women with 1–3 ANC contacts to 2.12 with 4–7 contacts and 2.41 with ≥8 contacts, demonstrating overlap between contact frequency and accumulated measured content.*
